# Which Aspects of Abortion Care Do Healthcare Practitioners in Britain Think Nurses/Midwives Should Provide? Findings From the SACHA Study

**DOI:** 10.1111/jan.70588

**Published:** 2026-03-29

**Authors:** Alice Fulton, Ona McCarthy, Jill Shawe, Rebecca Meiksin, Maria Lewandowska, Melissa Palmer, Rachel Scott, Kaye Wellings, Patricia Lohr, Geoffrey Wong, Sally Sheldon, Rebecca French and The SACHA Study Team, Annette Aronsson, Paula Baraitser, Sharon Cameron, Louise Keogh, Clare Murphy, Wendy V. Norman, Jennifer Reiter, Natasha Salaria

**Affiliations:** ^1^ London School of Hygiene and Tropical Medicine London UK; ^2^ University of Plymouth Plymouth UK; ^3^ British Pregnancy Advisory Service London UK; ^4^ University of Oxford Oxford UK; ^5^ University of Bristol Bristol UK

**Keywords:** Abortion, Midwives, Nurses, TBC

## Abstract

**Aim:**

To explore the views of healthcare practitioners in Britain regarding the role of midwives and nurses in the delivery of medical and surgical abortion.

**Design:**

An observational study of the Shaping Abortion for Change study healthcare practitioner survey (2021–2022).

**Methods:**

Relationships between healthcare practitioner type, participant characteristics, knowledge of and attitudes towards abortion, and views about nurses' and midwives' role in abortion care were examined using Pearson's Chi‐squared tests of association and multivariable logistic regression.

**Results:**

Amongst 763 participants including doctors, nurses, midwives and pharmacists, 71.6% supported specialist nurses in sexual and reproductive health and abortion clinics and hospitals, expanding their roles to include prescribing abortion medications and surgical abortion methods. Support was lower for midwives (35.8%) and primary care nurses (32.5%). There was considerable support for all nursing and midwifery groups to be involved in adjacent tasks of abortion care. Differences in support by healthcare practitioner type persisted after adjustment for exposure variables.

**Conclusion:**

There is strong support for specialist nurses to expand their role in abortion care. This change could be implemented following clarification of the legal position. Some healthcare practitioner groups are more reluctant to support broader involvement of nurses and midwives in abortion provision.

**Implications for the Profession and/or Patient Care:**

Expanding specialist nurses' role in abortion care could increase service capacity and improve patient access and experience. Understanding and addressing the concerns of healthcare practitioners opposing this change is critical for successful implementation and patient safety.

**Impact:**

This study addresses the potential for nurse and midwife role expansion in abortion care. The findings highlight broad support for specialist nurses whilst identifying barriers to wider role expansion. The research informs policy discussions on workforce optimisation and access to abortion services across Britain.

**Reporting Method:**

This study adheres to the STROBE guidelines for reporting observational studies.

**Patient or Public Involvement:**

In the SACHA study, patient and public involvement was included at all stages to inform study design, recruitment, data collection and analysis.

## 1 Introduction

In Britain, pursuant to the Abortion Act, an abortion must be approved and performed by doctors (Abortion Act 1967). The courts have interpreted ‘performed by a doctor’ to mean that a doctor must hold overall responsibility for all stages of the process, although they do not personally need to undertake all tasks (of Lords, G. B. E. H., of Appeal, C., and Division, C. 1981). This opens the possibility of the expansion of nurse and midwife involvement in provision of abortion services within the existing law. Moreover, change is afoot in the abortion care landscape. The Covid‐19 pandemic accelerated the role of telemedicine meaning that eligible patients can choose to undertake medical abortion at home without visiting a clinic, and, in light of changes to the law in Northern Ireland (decriminalisation of abortion and expansion of the role of nurses and midwives), it seems possible that future reform of abortion law in Britain would make specific provision for widening the range of health care professionals (HCPs) legally entitled to offer abortion services (Rooney and McGuinness 2020; French et al. 2022). Understanding HCP views on the future direction of abortion care in Britain, including the role of nurses and midwives, is thus of clear and pressing importance.

Currently in Britain, most abortion care is undertaken in specialist standalone clinics, mostly operated by third sector organisations such as the British Pregnancy Advisory Service (BPAS) and MSI Reproductive Choices. Nurses and midwives manage many of these services and undertake many aspects of abortion care, including providing counselling, dispensing medications, assisting in surgical procedures, and providing post abortion advice. However, they cannot approve abortion, prescribe abortion medications or, under the current dominant interpretation of the law, perform surgical abortion procedures. This prevents implementation of World Health Organisation (WHO) recommendations that nurses' and midwives' roles should be optimised to include prescribing medications and surgical abortion methods (Staerck 1967; World Health Organisation 2022; Zhou et al. 2020).

In 1981, the role of nurses and midwives in medical abortion care was unsuccessfully legally challenged, with the courts confirming that an abortion was ‘performed by’ a doctor and therefore lawful, where the doctor retained overall control of the procedure, even if not performing all tasks with his or her own hands (of Lords, G. B. E. H., of Appeal, C., and Division, C. 1981). On the basis of this ruling, it has been argued that appropriately trained nurses or midwives, acting as part of a multidisciplinary team that includes a doctor, could legally perform vacuum aspiration for surgical abortion, considering that some do exactly the same procedure following spontaneous miscarriage (Sheldon and Fletcher 2017). Nurses and midwives in Britain do act as medication prescribers following appropriate training in other settings, so expansion of their role to include prescribing abortion medications would fall within the accepted scope of practice.

Expanding nurses and midwives' roles in abortion care could expand settings in which abortion care can be provided, alleviate barriers to access and improve patient experience, as is the norm in many parts of the world (Zhou et al. 2020; Sheldon and Fletcher 2017; Qian et al. 2021; Munro et al. 2020; Endler et al. 2020; Sjostrom et al. 2016). Abortion was decriminalised in Northern Ireland in 2019 and the new legislation specifies that nurses and midwives can approve and perform abortions (Rooney and McGuinness 2020). In low and middle‐income countries (LMICs), ‘task‐shifting’ is widespread due to human resource constraints. In Canada, nurse practitioners are able to prescribe abortion medications (Canadian Institute for Health Information 2020). Professional bodies in Britain representing nurses and midwives (Royal College of Nursing (RCN) and Royal College of Midwives (RCM)) have released position statements advocating decriminalisation and the RCN 2024 abortion care guideline specifies that nurses’ roles should be developed to include manual vacuum aspiration (MVA) pending clarification of the legal position (Royal College of Nursing 2018, 2024; Royal College of Midwives 2023).

There is evidence from systematic reviews that, with training, medical and surgical abortion can be undertaken by nurses and midwives as safely and effectively as by doctors (Sjostrom et al. 2017; Barnard et al. 2015). The WHO's 2022 Abortion Care Guideline recommends that medical abortion and surgical abortion < 14 weeks can be undertaken without doctor involvement (World Health Organisation 2022).

HCPs' knowledge, attitudes and roles influence the implementation of expanded abortion care practices for nurses and midwives (Carvajal et al. 2022; Glenton et al. 2017). Studies suggest that HCPs with liberal views on abortion are more supportive of role expansion (Desai et al. 2022; Assefa 2019; Coleman‐Minahan et al. 2020). In multiple settings, doctors perceive nurses and midwives to lack the necessary skills required, despite evidence to the contrary (Carvajal et al. 2022; Glenton et al. 2017; Paul et al. 2014; Newton et al. 2016). HCP support for expanding the role of nurses and midwives in abortion care could influence policy and service development; improve the quality of training and could increase the confidence of those considering participating.

Research into British HCPs' attitudes towards nurses' and midwives' roles in abortion care is limited, with most studies focusing on doctors' views (French et al. 2023; Theodosiou and Mitchell 2015). The Shaping Abortion for Change (SACHA) study was established to provide evidence to help services and policymakers respond to the changing landscape in UK abortion care (Wellings 2023). As a component of this study, a survey of views and experiences of HCPs working within abortion and adjacent services was undertaken.

This paper explores the views of HCPs in Britain on the involvement of nurses and midwives in medical and surgical abortion care and examines how these views are shaped by characteristics, abortion knowledge, and attitudes to abortion.

## 2 Methodology

Full details of survey methods are described in detail elsewhere by French et al. (French et al. 2024). In brief, SACHA HCP survey data were collected between November 2021 and July 2022 (French et al. 2023). Doctors, nurses, midwives and pharmacists working in general practice, SRH clinics, pharmacies, maternity services (midwives only), and abortion services in England, Scotland and Wales were targeted. A stratified cluster sampling approach was used to ensure representation across regions, with systematic random sampling used to select services and then identify eligible staff members (French et al. 2023). The paper questionnaire was posted to individuals with the option to complete online.

Ethical approval for this data analysis was obtained from London School of Hygiene and Tropical Medicine (LSHTM) MSc Research Ethics committee on 15th May 2023 (Reference: 29190).

Exposure was defined as HCP type. Participants were categorised by role and service type to reflect variations in training and experience in abortion care. Doctors were divided into ‘GPs’ and ‘specialist doctors,’ the latter comprising those working in SRH clinics and abortion services. Nurses were classified as ‘primary care nurses’ (PCNs) or ‘specialist nurses’ (SNs), the latter working in SRH and abortion services. ‘Midwives’ were considered a single group as midwives are expected to provide non‐judgemental care and support informed choice, which could include discussion about abortion irrelevant of setting (The Royal College of Midiwives 2023; Nursing and Midwifery Council 2016). A final group comprised pharmacists.

The outcomes measured were HCPs' views on whether nurses and midwives should provide aspects of medical and surgical abortion care. The SACHA HCP survey asked: ‘with training, which other practitioners do you feel should be able to deliver the following aspects of abortion care?’ (see File 1.1). Aspects of care options that comprised delivery of abortion were used to construct outcome categories. Abortion care was categorised as either ‘active’ or ‘supportive’. ‘Active’ care was defined as an aspect of care performing either surgical or medical abortion. Under the current interpretation of British law, nurses and midwives are not permitted to undertake these tasks. These constituted: (i) prescribing abortion medications, (ii) carrying out surgical abortion up to 14 weeks gestation and (iii) carrying out surgical abortion at 14+ weeks gestation. Respondents advocating one of more of these tasks being undertaken by nurses or midwives were deemed supportive of active abortion care by that group.

‘Supportive’ care was defined as aspects of care that enable, facilitate or assist a medical or surgical abortion. These tasks are typically undertaken by nurses and midwives in specialist abortion units in the current configuration of British abortion services. These constituted: (i) dispensing or administering abortion medication, (ii) supporting women in abortion home management, (iii) discussing disposal of products of conception following home abortion, and (iv) inspection of products of conception to ensure completion. Pre and post abortion care were not addressed in this analysis. Again, respondents advocating one of more of these tasks being undertaken by nurses or midwives were deemed in favour of supportive abortion care by that group.

Six binary outcome categories were analysed: HCPs' views on whether midwives, PCNs or SNs should provide active or supportive abortion care. Each category of nurse or midwife and care type was considered separately to explore HCPs' views in detail (Table 1).

**TABLE 1:** Exposure and outcome variables.

Variable type	Variable	Categories
Exposure	Health care professional type (HCP type)	General practitioners (GPs) Specialist doctors Primary care nurses (PCNs) Specialist nurses (SNs) Midwives Pharmacists
Outcomes	Views on nurses and midwives providing aspects of abortion care	Midwives—active care^a^ Midwives—supportive care^b^ PCNs—active care PCNs—supportive care SNs—active care SNs—supportive care

^a^ Active care: prescribing abortion medications, performing surgical abortion < 14 weeks, performing surgical abortion > 14 weeks.

^b^ Supportive care: dispensing or administering abortion medications, supporting women in abortion home management, discussion disposal of products of conception following home abortion, inspection of products of conception to ensure completion.

Exposure variables comprised HCP characteristics that could influence views on abortion care as reported in the literature (Zhou et al. 2020; Glenton et al. 2017; Desai et al. 2022; Assefa 2019; Coleman‐Minahan et al. 2020; French et al. 2023; French and J. Wellings 2022; Dawson et al. 2016). These consisted of abortion provision status, age, gender, length of time qualified, religious beliefs, political beliefs, knowledge about abortion care/legislation and attitudes towards abortion. Knowledge was assessed using participants' responses to 6 statements relating to abortion law and practice guidelines with ‘true,’ ‘false’ and ‘don't know’ responses. Statements included ‘an abortion is a criminal offence unless it has been signed off by a doctor’ and ‘women must have an ultrasound before having a medical abortion’ (see File 1.2). Participants' responses were converted into a numerical score and divided into tertiles to represent low, medium, and high knowledge.

Attitudes to abortion were assessed using participants' responses to 7 statements on abortion including ‘abortion should not be carried out after 12 weeks gestation’ and ‘more medical abortions at earlier gestation may lead to more regretting their decision’ (see File 1.3). Participant responses were converted into a numerical score between 1 and 3 with ‘1’ representing the least liberal viewpoint and ‘3’ representing the most liberal viewpoint.

## 3 Data Analysis

All statistical analyses were undertaken using STATA/SE version 17.0 using the *surveyset* command to account for clustering by service.

Baseline characteristics and the distribution of variables were explored using descriptive statistics. Data were recoded where necessary, such as combining smaller gender categories. Associations between HCP type and views about which aspects of procedural abortion care nurses and midwives should provide were explored using design‐based Pearson Chi‐squared tests.

The influence of HCP characteristics, knowledge and attitudes on respective views about the provision of procedural care by nurses and midwives was explored by tabulating control variables by HCP type and each outcome category. Design‐based Pearson's Chi Squared tests were used to assess the strength of the association. A significance level of 5% was used to identify variables strongly associated with exposure or outcome. Exposure variables strongly associated with both exposure and outcome were identified as potential confounding variables and taken forward into multivariable logistic regression analyses.

Multivariable logistic regression analyses were undertaken. Crude and adjusted odds ratios (ORs) were calculated to assess the association between HCP type and beliefs about procedural abortion care, using GPs as a reference to facilitate comparison. Potential confounders were included in the model, and multicollinearity was checked to ensure model reliability. Adjusted Wald tests assessed the strength of associations.

## 4 Results

### 4.1 Characteristics of the Sample

Of 1370 HCPs surveyed, 771 (56.3%) completed the questionnaire. Amongst these, 763 participants who had complete data on role and the type of service they work in were included in this analysis.

Participants included nurses (34.2%), midwives (34.7%), doctors (23%) and pharmacists (8.1%). When categorised into groups combining role and service type (forming exposure variable ‘HCP type’), 11.5% of respondents were GPs, 11.4% specialist doctors, 7.6% were PCNs, 26.6% were SNs, 34.7% were midwives and 8.1% were pharmacists. Amongst midwives, 72.1% were from maternity services. Most of the remainder (25.3%) worked in abortion services. Overall, most respondents were female (87.6%), aged 40 years and above (61.8%), and secular in their religious views (68.1%). Nearly a third worked in abortion services (32.1%). A higher proportion of respondents (37.4%) had over 20 years' experience than had less than 5 years (12.7%). Political beliefs tended towards the left/left of centre (29.9%) or neutral (39.9%). Most participants had a high level of knowledge of abortion care (51.6%) and a liberal attitude towards abortion (attitude score 3, most liberal, 80.3%) (Table 2).

**TABLE 2:** Table of participant characteristics.

Variable	*n* (%)	95% CI
Role		
Doctor	176 (23.0)	17.2,30.2
Nurse	261 (34.2)	25.7,43.8
Midwife	265 (34.7)	21.6,50.6
Pharmacist	62 (8.1)	5.5,11.7
Service type		
SRH clinic	115 (14.9)	8.2,25.6
Maternity unit	198 (25.7)	13.3,43.8
GP practice	156 (20.3)	13.4,29.5
Pharmacy	54 (7)	4.6,10.4
Abortion service	247 (32.1)	21.2,45.3
Type of healthcare practitioner		
General practitioners	88 (11.5)	7.6,17.3
Specialist doctors	87 (11.4)	7.3,17.4
Primary care nurses	58 (7.6)	4.8,11.8
Specialist nurses	203 (26.6)	18.8,36.2
Midwives	265 (34.7)	21.6,50.6
Pharmacists	62 (8.1)	5.5,11.8
Age group		
Under 30	83 (11.0)	8.4,14.2
30–39	206 (27.2)	23.6,31.1
40–49	209 (27.6)	24.4,31
50+	260 (34.3)	29.6,39.3
Gender		
Female	662 (87.5)	83.4,90.6
Male	93 (12.3)	9.1,16.4
Other (non‐binary, other, prefer not to say)	2 (0.3)	0.07,1.0
Abortion provider		
No	293 (40.0)	32.6,47.9
Yes	439 (60.0)	52.1,67.4
Religion		
Very important	59 (7.8)	6.0,10.1
Quite important	147 (19.4)	16.7,22.6
Not important	515 (68.1)	63.8,72.1
Prefer not to say	35 (4.6)	3.4,6.3
Time since qualification		
Less than 5 years	96 (12.7)	9.8,16.3
5–10 years	163 (21.6)	18.1,25.4
11–20 years	214 (28.3)	25.2,31.6
Over 20 years	283 (37.4)	31.8,43.4
Politics		
Right/right of centre	30 (4.0)	2.7,5.7
Centre	112 (14.9)	12.5,17.6
Left/left of centre	225 (29.9)	26.4,33.7
None	300 (39.9)	35.7,44.4
Prefer not to say	85 (11.3)	8.9,14.26
Knowledge		
Low	103 (13.8)	10.5,17.9
Medium	257 (34.5)	28.7,40.7
High	386 (51.7)	43.6,59.7
Attitudes (1 = least liberal, 3 = most liberal)		
1	15 (2.0)	1.2,3.4
2	132 (17.7)	14.4,21.6
3	599 (80.3)	75.8, 84.1

Participant characteristics were explored by HCP type (see File 1.5). All participant characteristics were strongly associated with the exposure variable (all *p* values < 0.001).

Support for nurses and midwives providing active and supportive abortion was explored amongst all HCPs and by HCP type. Most HCPs (71.6%) supported SNs delivering active care. However, support for midwives (35.8%) or PCNs (32.5%) providing active care was limited amongst all (Table 3).

**TABLE 3:** Proportions of HCPs by type that think midwives, PCNs and SNs should provide active and supportive aspects of abortion care.

Healthcare practitioner	View that active abortion care should be provided by			View that supportive abortion care should be provided by		
	*n* (%)	95% CI	*p*	*n* (%)	95% CI	*p*
	Midwives			Midwives		
General practitioners	15 (17.1)	10.3, 27.0	0.002	37 (42.1)	32.7, 52.0	< 0.001
Specialist doctors	25 (28.7)	20.9, 38.1		68 (78.2)	67.3, 86.1	
Primary care nurses	17 (29.3)	17.8, 44.2		30 (51.7)	38.2, 65.0	
Specialist nurses	81 (39.9)	32.4, 47.9		137 (67.5)	57.9, 75.8	
Midwives	105 (39.6)	31.6, 48.2		220 (83.0)	73.2, 89.7	
Pharmacists	30 (48.4)	35.7, 61.3		53 (85.5)	72.3, 93.0	
All healthcare practitioners	273 (35.8)	31.9, 39.9		545 (71.4)	66.4, 76.0	

	Primary care nurses			Primary care nurses		
General practitioners	12 (13.6)	7.2, 24.3	0.001	41 (46.6)	33.0, 60.7	< 0.001
Specialist doctors	28 (32.2)	24.9, 40.4		72 (82.8)	74.9, 88.5	
Primary care nurses	17 (29.3)	17.5, 44.8		29 (50.0)	37.9, 62.1	
Specialist nurses	79 (38.9)	30.8, 47.7		146 (71.9)	64.4, 78.4	
Midwives	83 (31.3)	25.5, 37.8		199 (75.1)	70.8, 79.0	
Pharmacists	29 (46.8)	34.9, 59.0		53 (85.5)	75.1, 92.0	
All healthcare practitioners	248 (32.5)	28.7, 36.5		541 (70.8)	66.8, 74.4	

	Specialist nurses			Specialist nurses		
General practitioners	69 (78.4)	68.5, 85.8	0.002	84 (95.5)	88,6,98.3	0.002
Specialist doctors	67 (77.0)	69.1, 83.4		85 (97.7)	91.2, 99.4	
Primary care nurses	45 (77.6)	65.6, 86.3		49 (84.5)	72.9, 91.7	
Specialist nurses	155 (76.4)	70.2, 81.6		197 (97.0)	93.8, 98.6	
Midwives	165 (62.3)	54.8, 69.2		253 (95.5)	93.0, 97.1	
Pharmacists	45 (72.6)	60.9, 81.8		57 (91.9)	82.9, 96.4	
All healthcare practitioners	546 (71.6)	66.8, 75.9		727 (95.2)	93.3, 96.3	

Differences emerged when views were analysed by HCP type. GPs and PCNs were the least supportive of both midwives and PCNs providing any aspect of abortion care, whilst specialist doctors and SNs were more supportive. Conversely, pharmacists showed higher support for midwives (48.4%) and PCNs (46.8%) in active care roles than all other cadres of HCP. In relation to their own profession, 39.9% of midwives and 29.3% of PCNs were of the view that they should provide active care, whereas the majority of SNs (76.2%) were of the view that they should provide this service, a view shared by a majority amongst all other HCP cadres.

The view that nurses and midwives should provide supportive care was widely endorsed by all cadres of HCP, with 71.4% supporting midwives, 70.8% supporting PCNs, and 95.2% supporting SNs. Again, there were differences by HCP type; notably, less than 50% of GPs supported either midwives (42.1%) or PCNs (46.6%) providing supportive care, whilst 78.2% of specialist doctors supported midwives providing supportive care and 82.8% supported PCNs.

Participant characteristics were explored against outcome variables. Variables strongly associated with outcome were identified as confounders and included in the regression analysis. Significant differences in support for midwives, PCNs and SNs providing abortion care were identified according to participant characteristics (Table 4). Older participants were less supportive of midwives, with only 29.7% of those aged ≥ 50 endorsing midwives extending their practice to active roles compared with 39.1% of those < 30, and 61.2% supporting midwives in supportive roles compared with 88.1% of those < 30. A similar pattern emerged for PCNs. Conversely, older participants were the most supportive of SNs providing active care (74.2% vs. 56% of < 30s).

**TABLE 4:** Participant characteristics by views about which aspects of abortion care midwives and nurses should provide.

	Views supportive of midwives		Views supportive of primary care nurses		Views supportive of specialist nurses	
	Active abortion care	Supportive abortion care	Active abortion care	Supportive abortion care	Active abortion care	Supportive abortion care
	*n* (%) (95% CI)	*n* (%) (95% CI)	*n* (%) (95% CI)	*n* (%) (95% CI)	*n* (%) (95% CI)	*n* (%) (95% CI)
Age group	*	**	—	*	*	*
Under 30	32 (39.1) (29.2, 50.4)	73 (88.1) (78.9, 93.6)	28 (33.3) (24.4, 43.7)	72 (86.9) (76.8, 93.0)	46 (56.0) (45.4, 66.0)	82 (98.8) (91.6, 99.8)
30–39	84 (40.9) (34.6, 47.5)	157 (76.0) (69.1, 81.7)	67 (32.7) (26.1, 40.0)	150 (72.6) (64.6, 79.4)	152 (73.6) (66.7, 79.4)	202 (98.1) (95.3, 99.2)
40–49	79 (37.8) (30.4, 45.8)	157 (75.1) (68.1, 81.0)	66 (31.6) (25.3, 38.7)	146 (69.7) (62.6, 76.2)	153 (73.2) (65.5, 79.7)	200 (95.7) (92.3, 97.6)
50+	77 (29.7) (24.9, 35.0)	159 (61.2) (54.6, 67.5)	86 (33.1) (27.5, 39.2)	172 (66.2) (60.5, 71.4)	193 (74.2) (68.0, 79.5)	239 (92.0) (88.1, 94.7)
Gender	—	—	—	—	—	—
Female	241 (36.4) (32.2, 40.8)	483 (72.9) (67.3, 77.9)	216 (32.6) (28.7, 36.9)	469 (70.8) (66.8, 74.5)	467 (70.5) (65.3, 75.3)	630 (95.1) (93.2, 96.4)
Male	31 (33.3) (24.5, 43.5)	61 (65.6) (54.9, 74.9)	31 (33.3) (24.8, 43.1)	68 (73.1) (61.7, 82.1)	74 (79.6) (70.6, 86.4)	92 (98.9) (92.7, 99.9)
Other	1 (50.0) (5.7, 94.3)	0	0	2 (100)	0	0
Abortion provider	*	*	—	*	—	*
No	93 (31.6) (26.5, 37.3)	182 (62.2) (55.7, 68.4)	91 (31.0) (25.9, 36.5)	193 (66.0) (59.3, 72.1)	206 (70.4) (64.0, 76.1)	274 (93.4) (90.6, 95.6)
Yes	169 (38.5) (33.6, 43.7)	342 (77.9) (71.0, 83.6)	146 (33.3) (28.5, 38.5)	326 (74.3) (69.6, 78.5)	319 (72.6) (67.4, 77.5)	423 (96.4) (92.3, 97.8)
Religion	*	*	—	—	—	*
Very important	17 (28.3) (18.5, 40.8)	39 (66.7) (53.5, 77.7)	21 (35.0) (23.1, 49.1)	40 (68.3) (54.8, 79.4)	42 (71.7) (59.4, 81.4)	53 (90.0) (80.6, 95.1)
Quite important	38 (25.9) (20.0, 32.8)	93 (63.3) (54.4, 71.3)	40 (27.2) (20.5, 35.1)	103 (70.1) (61.6, 77.4)	102 (69.4) (62.1, 75.8)	143 (97.3) (92.7, 99.0)
Not important	210 (40.7) (35.8, 45.8)	390 (75.7) (69.9, 80.8)	176 (34.1) (29.8, 38.7)	375 (72.8) (68.1, 77.1)	377 (73.2) (67.3, 78.4)	495 (96.2) (94.1, 97.5)
Prefer not to say	10 (27.8) (15.5, 44.7)	23 (66.7) (48.9, 80.7)	11 (30.6) (17.6, 47.6)	21 (61.1) (44.3, 76.7)	22 (63.9) (46.9, 78.0)	31 (88.9) (74.7, 95.6)
Time since qualification	*	**	—	*	*	**
Less than 5 years	37 (38.1) (30.3, 46.7)	81 (84.5) (76.3, 90.3)	32 (33.0) (25.8, 41.0)	76 (79.4) (68.7, 87.1)	54 (56.7) (46.8, 66.1)	94 (97.9) (91.8, 99.5)
5–10 years	69 (42.2) (34.6, 50.2)	129 (78.9) (70.5, 85.4)	55 (34.0) (27.5, 43.2)	128 (78.3) (70.3, 84.6)	118 (72.3) (64.2, 79.2)	159 (97.6) (94.1, 99.0)
11–20 years	87 (40.5) (33.5, 47.8)	165 (77.2) (69.7, 83.3)	70 (32.6) (25.5, 40.5)	154 (72.1) (65.8, 77.6)	160 (74.9) (66.7, 81.6)	210 (98.1) (92.3, 99.3)
Over 20 years	81 (28.5) (23.3, 34.3)	168 (59.5) (52.8, 65.8)	89 (31.3) (26.3, 36.8)	179 (63.4) (57.3, 69.1)	210 (74.3) (68.7, 79.2)	258 (91.2) (87.3, 94.0)
Politics	—	—	—	—	—	**
Right/right of centre	12 (40.0) (22.5, 60.5)	19 (63.3) (44.0, 79.1)	15 (50.0) (30.7, 69.3)	22 (73.3) (49.2, 88.6)	26 (86.7) (65.5, 95.7)	27 (90.0) (74.2, 96.6)
Centre	33 (29.2) (21.3, 38.6)	74 (66.4) (57.6, 74.1)	34 (30.1) (22.1, 39.5)	75 (67.3) (58.4, 75.0)	85 (76.1) (68.0, 82.7)	109 (97.4) (91.9, 99.2)
Left/left of centre	85 (37.9) (32.3, 43.8)	176 (78.0) (71.4, 83.4)	66 (29.5) (23.5, 36.3)	171 (75.8) (69.2, 81.4)	168 (74.5) (61.4, 75.6)	225 (100)
None	118 (39.3) (33.3, 45.6)	213 (71.0) (63.8, 77, 2)	105 (35.0) (28.9, 41.6)	226 (75.3) (67.4, 76.7)	207 (69.0) (61.4, 75.6)	298 (94.4) (90.8, 96.6)
Prefer not to say	25 (29.1) (19.8, 40.5)	60 (70.9) (58.7, 80.7)	26 (30.2) (20.5, 42.2)	51 (60.5) (48.6, 71.2)	56 (66.3) (56.0, 75.3)	75 (88.4) (80.7, 93.2)
Knowledge	—	—	—	—	—	—
Low	37 (35.6) (26.7, 45.5)	74 (72.1) (61.6, 80.7)	26 (25.0) (17.9, 33.7)	68 (66.4) (58.5, 73.4)	70 (68.3) (58.9, 76.3)	96 (93.3) (87.7, 96.4)
Medium	88 (34.2) (28.7, 40.3)	174 (67.7) (60.6, 74.1)	86 (33.5) (27.6, 39.0)	183 (71.2) (65.1, 76.5)	179 (69.6) (61.9, 76.4)	242 (94.2) (90.8, 96.4)
High	144 (37.4) (31.8, 43.3)	288 (74.5) (67.8, 80.2)	133 (34.5) (29.2, 40.3)	279 (72.2) (67.5, 76.4)	286 (74.0) (69.2, 78.3)	371 (96.1) (93.9, 97.6)
Attitude (1 = least liberal, 3 = most liberal)	*	**	—	**	—	**
1	2 (13.3) (3.3, 41.0)	5 (33.3) (14.3, 59.7)	2 (13.3) (3.3, 41.0)	7 (46.7) (23.9, 71.0)	7 (46.7) (23.9, 71.0)	10 (66.7) (40.3, 85.6)
2	30 (22.7) (15.6, 32.0)	67 (50.8) (41.5, 60.0)	33 (25.0) (17.2, 34.9)	70 (53.0) (42.5, 63.3)	91 (68.9) (59.1, 77.3)	124 (93.9) (88.5, 96.9)
3	234 (39.1) (34.5, 43.9)	462 (77.1) (71.9, 81.6)	207 (34.6) (30.2, 39.3)	453 (75.6) (72.2, 78.8)	433 (72.3) (68.2, 77.9)	579 (96.7) (94.7, 97.9)

*Note:* Grey shade *p* value does not meet acceptable significance level for this study or *p* > 0.05.

* *p* ≤ 0.05.

** *p* < 0.001.

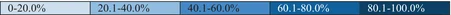

Abortion providers were significantly more supportive than non‐providers, for example, endorsing midwives in active (38.5% vs. 31.6%) and supportive roles (77.9% vs. 62.2%). Religion was associated with views about midwives and views about supportive care by SNs. Participants reporting religion as ‘not important’ were most supportive of midwives (active 40.7%), while those reporting religion as ‘quite important’ were most supportive of SNs in supportive care (97.3%).

Time since qualification was associated with views about midwives, SNs and supportive care by PCNs. In general, those qualified for longer were less supportive of extending roles than those more recently qualified. Amongst those qualified for > 20 years, 28.5% advocated active care by midwives vs. 42.2% of those qualified for 5–10 years. Liberal attitudes were consistently associated with greater support in most groups (e.g., midwives in supportive care: 77.1% vs. 33.3% amongst the least liberal).

Following multivariable analysis, a significant association was identified between HCP type and views about midwives, supportive care by PCNs and supportive care by SNs. Adjustment did not change the direction of association (see File 1.6).

## 5 Discussion

Amongst HCPs surveyed, support for SNs undertaking both active and supportive abortion care roles was widespread. Support for midwives or PCNs delivering active aspects of abortion care was limited, although supportive care was widely endorsed. This finding may reflect the structure of abortion services in the UK (in standalone clinics and most closely aligned to SRH services) indicating that nurses with an SRH skillset are most suited to role expansion. The reluctance to expand the roles of midwives and PCNs to include active abortion care may stem from a lack of exposure in these roles to abortion, meaning that HCPs do not view midwives or PCNs as having the competence or confidence to perform these tasks. This result contrasts with findings in Sweden where the status of midwives, and their more life course approach to the provision of reproductive care, was felt to be integral to the success of task distribution in abortion care (Endler et al. 2020).

Less than half of midwife respondents supported their role expansion to active abortion care. Most midwives surveyed work in maternity units where supportive abortion care is routine practice, although this is generally performed in the second trimester for medical reasons. Although midwives in maternity units may be familiar with abortion for medical reasons, they lack exposure and may be uncomfortable with abortion processes at earlier gestations. Additionally, the RCM abortion position statement describes a midwives' scope of practice to include ‘work with women’ considering abortion, whereas RCN guidance is more robust about the potential for nurses' skillset to include independent abortion practice (Royal College of Nursing 2024; Royal College of Midwives 2023). This finding contradicts those of studies outside of the UK, in Ethiopia, and USA, where being a midwife was associated with interest in extending skills in abortion (Assefa 2019; Coleman‐Minahan et al. 2020).

Low proportions of GPs and PCNs supported PCN involvement in either active or supportive abortion care compared to other HCPs. This suggests that primary care practitioners view abortion care as less appropriate in primary care settings, but the reasons for this association are unclear. A qualitative Canadian study investigating underutilisation of nurse‐provided medical abortion in primary care settings found participants who did not provide medical abortion felt abortion was a specialised service and outside the remit of primary care. A lack of education and mentorship were identified as barriers to providing abortion services in this setting (Carson et al. 2023). In an Australian study, GPs doubted the ability of PCNs to provide early medical abortion (Newton et al. 2016). However, these studies were undertaken in different healthcare systems; hence, it would be crude to draw inference from these findings. One possible explanation is that the survey took place during a period of increasing workforce pressure and workload in primary care in Britain. Primary care practitioners may have been expressing their reluctance to take on additional work without appropriate funding.

Specialist doctors and SNs demonstrated more support for sharing abortion care tasks with midwives and PCNs than their primary care counterparts. This aligns with previous research indicating that HCPs familiar with abortion care are more comfortable with procedures occurring outside specialised services (Carson et al. 2023; Michie et al. 2013).

A higher proportion of pharmacists supported midwives and PCNs in both active and supportive abortion care roles compared to other HCPs. This finding should be interpreted with caution due to data scarcity; however, in recent years pharmacists have taken on SRH roles such as over‐the‐counter emergency contraception and oral contraceptive pill prescription meaning they may be more amenable to distributing tasks in abortion care.

Younger and less experienced HCPs were more likely to support midwives in active and supportive roles, whilst older, more experienced HCPs were more supportive of SNs providing active abortion care, suggesting a generational shift in attitudes. In other studies, younger and more recently qualified practitioners were also more likely to advocate expanding roles in abortion care (Desai et al. 2022; Assefa 2019; Coleman‐Minahan et al. 2020). Older and more experienced practitioners may have trained at a time when independent practice was less widespread, and they may lack knowledge about how nurses' and midwives' roles in abortion care have evolved.

Those providing abortion care were more likely to support role expansion for nurses and midwives. Those who are more familiar with what abortion care entails were more confident that other types of providers could provide this too. This echoes findings from a survey of HCPs' views on telemedicine for abortion, in which providers who have been involved in telemedicine were more supportive of its ongoing use (Meiksin et al. 2024). Additionally, those with more liberal attitudes towards abortion were more likely to support role expansion for nurses and midwives. This corresponds with several previous HCP surveys in the UK and internationally (Desai et al. 2022; Coleman‐Minahan et al. 2020; Theodosiou and Mitchell 2015). Systematic review findings identifying positive socio‐cultural attitudes towards abortion as a facilitator of task‐shifting policies in abortion care appear to be consistent in the UK context (Zhou et al. 2020).

## 6 Strengths and Limitations of the Work

The sampling strategy was designed to maximise the ability to achieve a representative sample of HCPs working in the target services in Britain; however, the response rate amongst those sent the questionnaire was moderate at 56.3% (French et al. 2023). Some HCP types were underrepresented (e.g., pharmacists and PCNs), affecting the generalisability of the findings. Other professional groups who are or could be involved in abortion care, such as gynaecology doctors and nurses/midwives in early pregnancy settings, were not surveyed. Despite this, the inclusion of a broad range of HCPs from different services makes this survey one of the largest and most comprehensive of its kind. Respondents were asked about factors known to be associated with views on abortion, meaning residual confounding is unlikely. Selection bias may be a concern as most respondents were involved in abortion care, potentially skewing the results.

## 7 Implications for Policy and Practice

This study offers insights into HCPs' views about the role of nurses/midwives in both active and supportive aspects of abortion care and identifies groups considered more suited to innovations in current policy and practice. Research has consistently shown that abortion delivered by nurses and midwives is as safe as that delivered by doctors (Sjostrom et al. 2017; Barnard et al. 2015). It is highly acceptable to women, can reduce waiting times, improve patient experience and offer more patient choice (Endler et al. 2020; Sjostrom et al. 2016; Barnard et al. 2015). This study identifies broad support from HCPs for the implementation of WHO recommendations to optimise the roles of nurses and midwives in supportive abortion care, and suggests policymakers should expand the role of SNs to include prescribing abortion medication and, initially, surgical abortion < 14 weeks (World Health Organisation 2022; Footman 2023). With clarification, this could be undertaken within the current legal framework (Sheldon and Fletcher 2017).

Successfully expanding the roles of nurses and midwives in abortion care to include prescribing abortion medications and surgical abortion must be sensitive to embedded structures whilst encouraging a collaborative culture. Buy‐in from professional bodies representative of nurses and midwives (RCM and RCN) is essential. The expansion of nurses and midwives' roles must be considered relevant to their professional scope of practice and should not be undertaken to fill gaps due to a lack of doctors. To address workforce issues, particularly in surgical abortion, skilled personnel of all cadres are required.

Ongoing research is needed. Qualitative research should explore the perspectives, challenges and motivations of primary care practitioners in relation to the expanded roles of nurses and midwives and the delivery of abortion care in primary care settings. The views of pharmacists as potential providers of medical abortion also require exploration.

## 8 Conclusion

This research identifies differences in HCPs' views about the roles of nurses and midwives in abortion care. Support for SNs to extend their roles to include prescribing abortion medications and surgical abortion was high amongst all surveyed HCPs. Arguably, this change could be implemented within the current interpretation of the law. This needs urgent clarification. There was less support for midwives and PCNs in expanded roles, although supportive abortion care was widely endorsed. The findings reflect the structure of abortion services in the UK and indicate that HCPs' perceptions are influenced by personal characteristics and values. Addressing these dynamics through dialogue, education, and collaboration will be essential for achieving patient‐centred and evidence‐based abortion care services.

## Author Contributions

K.W. and R.F. conceptualised the study. R.F., M.P. and J.S. designed the survey and materials, with input from all authors. R.F., J.S., M.P., R.M., and N.S. managed ethics/R&D approvals and fieldwork. A.F. conducted the analysis as part of her MPH research project at LSHTM, supervised by O.M. A.F. drafted the paper with input from O.M., J.S., M.P., R.M., R.S., M.L., P.L., G.W., S.S. and K.W. All authors have read and approved the final manuscript.

## Funding

This paper is a part of the SACHA Study, funded by the National Institute of Health Research, award NIHR129529.

## Ethics Statement

Approval was obtained from the London School of Hygiene and Tropical Medicine ethics committee (ref: 29190).

## Conflicts of Interest

The authors declare no conflicts of interest.

## Supporting information


**Data S1:** Supporting Information.

**Data S2:** Supporting Information.

## Data Availability

Data are not available; it is confidential and subject to ethical approvals.
