# Supplementary material for: Which Aspects of Abortion Care Do Healthcare Practitioners in Britain Think Nurses/Midwives Should Provide? Findings From the SACHA Study
Source: J Adv Nurs. 2026 Mar 29;82(11):9796–806. doi: 10.1111/jan.70588 (PMC13661790; doi:10.1111/jan.70588)
Supplement: Supplementary file 1 [file JAN-82-9796-s001.docx]

# Supplementary file

1.1: Question in SACHA survey used to generate data about HCPs’ beliefs about which aspects of procedural care midwives and nurses should provide

1.2: Statements in the SACHA survey used to generate knowledge score

1.3: Statements in SACHA survey used to generate attitudes score

1.4a: Table of participant characteristics

| **Variable** | **n (%)** | **95% CI** |
| --- | --- | --- |
| **Role** |  |  |
| Doctor | 176 (23.0) | 17.2,30.2 |
| Nurse | 261 (34.2) | 25.7,43.8 |
| Midwife | 265 (34.7) | 21.6,50.6 |
| Pharmacist | 62 (8.1) | 5.5,11.7 |
| **Service Type** |  |  |
| SRH clinic | 115 (14.9) | 8.2,25.6 |
| Maternity Unit | 198 (25.7) | 13.3,43.8 |
| GP practice | 156 (20.3) | 13.4,29.5 |
| Pharmacy | 54 (7) | 4.6,10.4 |
| Abortion service | 247 (32.1) | 21.2,45.3 |
| **Type of healthcare practitioner** |  |  |
| General practitioners | 88 (11.5) | 7.6,17.3 |
| Specialist doctors | 87 (11.4) | 7.3,17.4 |
| Primary care nurses | 58 (7.6) | 4.8,11.8 |
| Specialist nurses | 203 (26.6) | 18.8,36.2 |
| Midwives | 265 (34.7) | 21.6,50.6 |
| Pharmacists | 62 (8.1) | 5.5,11.8 |
| **Age group** |  |  |
| Under 30 | 83 (11.0) | 8.4,14.2 |
| 30-39 | 206 (27.2) | 23.6,31.1 |
| 40-49 | 209 (27.6) | 24.4,31 |
| 50+ | 260 (34.3) | 29.6,39.3 |
| **Gender** |  |  |
| Female | 662 (87.5) | 83.4,90.6 |
| Male | 93 (12.3) | 9.1,16.4 |
| Other (Non-binary, other, prefer not to say) | 2 (0.3) | 0.07,1.0 |
| **Abortion provider** |  |  |
| No | 293 (40.0) | 32.6,47.9 |
| Yes | 439 (60.0) | 52.1,67.4 |
| **Religion** |  |  |
| Very important | 59 (7.8) | 6.0,10.1 |
| Quite important | 147 (19.4) | 16.7,22.6 |
| Not important | 515 (68.1) | 63.8,72.1 |
| Prefer not to say | 35 (4.6) | 3.4,6.3 |
| **Time since qualification** |  |  |
| Less than 5 years | 96 (12.7) | 9.8,16.3 |
| 5-10 years | 163 (21.6) | 18.1,25.4 |
| 11-20 years | 214 (28.3) | 25.2,31.6 |
| Over 20 years | 283 (37.4) | 31.8,43.4 |
| **Politics** |  |  |
| Right/ right of centre | 30 (4.0) | 2.7,5.7 |
| Centre | 112 (14.9) | 12.5,17.6 |
| Left/ left of centre | 225 (29.9) | 26.4,33.7 |
| None | 300 (39.9) | 35.7,44.4 |
| Prefer not to say | 85 (11.3) | 8.9,14.26 |
| **Knowledge** |  |  |
| Low | 103 (13.8) | 10.5,17.9 |
| Medium | 257 (34.5) | 28.7,40.7 |
| High | 386 (51.7) | 43.6,59.7 |
| **Attitudes (1= least liberal, 3=most liberal)** |  |  |
| 1 | 15 (2.0) | 1.2,3.4 |
| 2 | 132 (17.7) | 14.4,21.6 |
| 3 | 599 (80.3) | 75.8, 84.1 |

1.4b: Table of participants by role and service type

|  | **Type of service (n) (%, 95%CI)** | | | | |
| --- | --- | --- | --- | --- | --- |
| **Role** | **SRH clinic** | **Maternity unit** | **GP practice** | **Pharmacy** | **Abortion service** |
| **Doctor** | 23 (13.1%, 6.7–23.9) | 0 (0%) | 89 (50.6%, 35.5–65.5) | 0 (0%) | 64 (36.4%, 22.0–53.6) |
| **Nurse** | 89 (34.1%, 20.3–51.3) | 2 (0.8%, 0.1–5.4) | 58 (22.2%, 13.8–33.7) | 0 (0%) | 112 (42.9%, 28.5–58.7) |
| **Midwife** | 1 (0.4%, 0.05–2.9) | 191 (72.1%, 45.4–88.9) | 6 (2.3%, 0.5–9.7) | 0 (0%) | 67 (25.3%, 9.5–52.1) |
| **Pharmacist** | 2 (3.2%, 0.8–12.3) | 1 (1.6%, 0.2–10.9) | 3 (4.8%, 1.1–18.7) | 54 (87.1%, 73.0–94.4) | 2 (3.2%, 0.4–19.9) |
