# Supplementary material for: Which Aspects of Abortion Care Do Healthcare Practitioners in Britain Think Nurses/Midwives Should Provide? Findings From the SACHA Study
Source: J Adv Nurs. 2026 Mar 29;82(11):9796–806. doi: 10.1111/jan.70588 (PMC13661790; doi:10.1111/jan.70588)
Supplement: Supplementary file 2 [file JAN-82-9796-s002.docx]

# Supplementary file

1.1: Question in SACHA survey used to generate data about HCPs’ beliefs about which aspects of procedural care midwives and nurses should provide

1.2: Statements in the SACHA survey used to generate knowledge score

1.3: Statements in SACHA survey used to generate attitudes score

1.4: Table of participants by role and service type

|  | **Type of service (n) (%, 95%CI)** | | | | |
| --- | --- | --- | --- | --- | --- |
| **Role** | **SRH clinic** | **Maternity unit** | **GP practice** | **Pharmacy** | **Abortion service** |
| **Doctor** | 23 (13.1%, 6.7–23.9) | 0 (0%) | 89 (50.6%, 35.5–65.5) | 0 (0%) | 64 (36.4%, 22.0–53.6) |
| **Nurse** | 89 (34.1%, 20.3–51.3) | 2 (0.8%, 0.1–5.4) | 58 (22.2%, 13.8–33.7) | 0 (0%) | 112 (42.9%, 28.5–58.7) |
| **Midwife** | 1 (0.4%, 0.05–2.9) | 191 (72.1%, 45.4–88.9) | 6 (2.3%, 0.5–9.7) | 0 (0%) | 67 (25.3%, 9.5–52.1) |
| **Pharmacist** | 2 (3.2%, 0.8–12.3) | 1 (1.6%, 0.2–10.9) | 3 (4.8%, 1.1–18.7) | 54 (87.1%, 73.0–94.4) | 2 (3.2%, 0.4–19.9) |

|  | **General Practitioners** | | **Specialist doctors** | | **Primary care nurses** | | **Specialist nurses** | | **Midwives** | | **Pharmacists** | |  |
| --- | --- | --- | --- | --- | --- | --- | --- | --- | --- | --- | --- | --- | --- |
|  | n (%) | 95% CI | n (%) | 95% CI | n (%) | 95% CI | n (%) | 95% CI | n (%) | 95% CI | n (%) | 95% CI | *p* |
| **Age group** | | | | | | | | | | | | | |
| Under 30 | 1 (1.2) | 0.2,8.5 | 2 (2.4) | 0.58,9.6 | 1 (1.2) | 0.15,8.5 | 20 (24.1) | 12.8,40.8 | 45 (54.2) | 34.3,72.9 | 14 (16.9) | 8.9,29.6 | <0.001 |
| 30-39 | 24 (11.7) | 6.7,19.6 | 26 (12.6) | 6.5,23.0 | 10 (4.9) | 2.3,10.1 | 54 (26.2) | 16.5,38.9 | 77 (37.4) | 21.6,56.4 | 15 (7.3) | 3.9,13.1 |  |
| 40-49 | 32 (15.3) | 9.6,23.6 | 24 (11.48) | 6.7,19.1 | 14 (6.7) | 3.4,12.8 | 50 (23.9) | 16.1,34.0 | 71 (34.0) | 21.5,49.2 | 18 (8.6) | 5.2,14.02 |  |
| 50+ | 31 (11.9) | 7.3,18.7 | 34 (13.1) | 8.9,18.9 | 32 (12.3) | 7.2,20.2 | 79 (30.4) | 21.4,41.1 | 70 (27.0) | 15.2,43.1 | 14 (5.4) | 2.9,9.7 |  |
| **Gender** | | | | | | | | | | | | | |
| Female | 46 (6.9) | 4.3,11.1 | 65 (9.8) | 6.1,15.5 | 58 (8.8) | 5.4,13.9 | 196 (29.6) | 20.6,40.6 | 257 (38.8) | 24.4,55.5 | 40 (6.0) | 3.9,9.3 | <0.001 |
| Male | 41 (44.1) | 31.1,57.9 | 21 (22.6) | 13.3,35.8 | 0 (0.0) |  | 7 (7.5) | 3.6,14.9 | 3 (3.2) | 1.0,9.7 | 21 (22.6) | 14.3,33.8 |  |
| Other | 0 (0.0) |  | 0 (0.0) |  | 0 (0.0) |  | 0 (0.0) |  | 2 (100.0) |  | 0 (0.0) |  |  |
| **Abortion provider** | | | | | | | | | | | | | |
| No | 39 (13.3) | 8.3,20.5 | 12 (4.1) | 2.1,7.7 | 49 (16.7) | 10.5,25.4 | 39 (13.3) | 7.6,22.0 | 97 (33) | 17.8,52.9 | 57 (19.5) | 13.0,28.1 | <0.001 |
| Yes | 47 (10.7) | 6.2,17.7 | 72 (16.4) | 10.5,24.8 | 8 (1.8) | 0.8,4.1 | 152 (34.6) | 24.7,46.1 | 155 (35.3) | 21.8,51.7 | 5 (1.1) | 0.4,3.3 |  |
| **Religion** | | | | | | | | | | | | | |
| Very important | 11 (18.6) | 9.4,33.5 | 7 (11.9) | 5.6,23.5 | 7 (11.9) | 5.1,25.2 | 6 (10.2) | 3.7,25.3 | 16 (27.2) | 12.8,48.5 | 12 (20.3) | 11.2,34.0 | <0.001 |
| Quite important | 27 (18.4) | 11.0,29.2 | 16 (10.9) | 5.9,19.1 | 10 (6.8) | 3.2,14.0 | 31 (21.1) | 12.8,32.7 | 47 (32.0) | 16.8,52.3 | 16 (10.9) | 6.2,18.4 |  |
| Not important | 46 (8.9) | 5.5,14.1 | 58 (11.3) | 6.7,18.3 | 38 (7.4) | 4.5,11.9 | 154 (30) | 20.9,40.7 | 190 (36.9) | 23.7,52.3 | 29 (5.6) | 3.5,9.0 |  |
| Prefer not to say | 4 (11.4) | 4.4,26.7 | 5 (14.3) | 5.9,30.7 | 3 (8.6) | 2.8,23.3 | 12 (34.3) | 19.0,53.7 | 7 (20.0) | 8.9,39.2 | 4 (11.4) | 4.2,27.3 |  |
| **Time since qualification** | | | | | | | | | | | | | |
| Less than 5 years | 5 (5.2) | 2.0, 12.9 | 5 (5.2) | 1.4,17.7 | 2 (2.1) | 0.5,8.5 | 25 (26.0) | 13.7,43.8 | 48 (50.0) | 30.1,70.0 | 11 (11.5) | 5.5,22.4 | <0.001 |
| 5-10 years | 18 (11.0) | 5.7,20.5 | 10 (6.1) | 2.3,15.1 | 7 (4.3) | 1.5,11.6 | 34 (20.9) | 12.3,33.19 | 82 (50.3) | 33.5,67.0 | 12 (7.4) | 3.8,13.7 |  |
| 11-20 years | 24 (11.2) | 6.6,21.8 | 29 (13.6) | 8.1,21.7 | 14 (6.5) | 4.3,12.2 | 61 (28.5) | 19.6,39.4 | 69 (32.2) | 18.6,49.8 | 17 (7.9) | 4.5,13.5 |  |
| Over 20 years | 41 (14.5) | 9.3,21.8 | 42 (14.8) | 10.1,21.4 | 35 (12.4) | 7.7,19.2 | 82 (29.0) | 20.7,39 | 62 (21.9) | 11.5,37.7 | 21 (7.4) | 4.4,12.4 |  |
| **Politics** | | | | | | | | | | | | | |
| Right/ right of centre | 5 (16.7) | 6.4,37.2 | 3 (10.0) | 3.1,27.6 | 6 (20.0) | 9.4,37.5 | 5 (16.7) | 6.0,38.7 | 5 (16.7) | 5.3,41.7 | 6 (20.0) | 8.8,39.3 | <0.001 |
| Centre | 27 (24.1) | 15.7,35.1 | 13 (11.6) | 6.5,19.9 | 6 (5.4) | 2.4,11.4 | 27 (24.1) | 14.7,37 | 27 (24.1) | 11.9,42.6 | 12 (10.7) | 5.9,18.7 |  |
| Left/ left of centre | 36 (16.0) | 10,24.7 | 40 (17.8) | 10.2,29.1 | 11 (4.9) | 2.3,10.1 | 44 (19.6) | 13.0,28.3 | 82 (36.4) | 23.1,52.3 | 12 (5.3) | 2.9,9.6 |  |
| None | 11 (3.6) | 1.8,7.3 | 19 (6.3) | 3.5,11.1 | 24 (8.0) | 4.3,14.3 | 13 (4.2) | 22.8,47.8 | 122 (40.5) | 25.1,58.1 | 22 (7.3) | 4.3,12.0 |  |
| Prefer not to say | 8 (9.4) | 4.4,19.2 | 11 (12.9) | 6.8,23.3 | 10 (11.8) | 5.5,23.6 | 23 (27.1) | 16.9,40.4 | 24 (28.2) | 16.0,44.9 | 9 (10.6) | 5.3,19.9 |  |
| **Knowledge** | | | | | | | | | | | | | |
| Low | 13 (12.6) | 6.7,22.4 | 2 (1.9) | 0.5,7.8 | 19 (18.5) | 9.8,31.8 | 5 (4.9) | 1.6,13.5 | 39 (37.9) | 20.0,59.7 | 25 (24.3) | 14.5,37.7 | <0.001 |
| Medium | 44 (17.1) | 10.5,26.8 | 5 (1.9) | 0.8,4.7 | 22 (8.6) | 5.1,14.1 | 52 (20.2) | 12.4,31.3 | 111 (43.2) | 26.3,61.8 | 23 (8.9) | 5.1,15.3 |  |
| High | 26 (6.7) | 4.0,11.1 | 79 (20.5) | 13.9,29.1 | 14 (3.6) | 1.8,7.2 | 140 (36.3) | 27.2,46.4 | 113 (29.3) | 17.7,44.4 | 14 (3.6) | 2.0,6.6 |  |
| **Attitude (1= least liberal, 3=most liberal)** | | | | | | | | | | | | | |
| 1 | 4 (26.7) | 10.3,53.7 | 0 (0.0) |  | 4 (26.7) | 10.3,53.7 | 1 (6.7) | 0.9,35.8 | 2 (13.3) | 3.3,41.0 | 4 (26.7) | 10.3,53.7 | <0.001 |
| 2 | 28 (21.2) | 13.6,31.6 | 8 (6.1) | 2.9,12.0 | 22 (16.7) | 10.2,26.0 | 18 (13.6) | 13.6,23.1 | 41 (31.1) | 17.5,48.9 | 15 (11.4) | 6.4,19.4 |  |
| 3 | 55 (9.2) | 5.8,17.3 | 77 (12.9) | 5.8,14.3 | 32 (5.3) | 3.1,9.1 | 178 (29.7) | 21.0,40.2 | 217 (36.2) | 22.5,52.7 | 40 (6.7) | 4.3,10.2 |  |

1.5 The characteristics of participants by type of HCP

Percentages: row P values: design-based Pearson’s Chi Squared test.

|  | **View that aspects of active abortion care should be provided by:** | | | | | | **View that supportive abortion care should be provided by:** | | | | | |
| --- | --- | --- | --- | --- | --- | --- | --- | --- | --- | --- | --- | --- |
|  | Midwives | | | | | | Midwives | | | | | |
|  | OR | 95% CI | *p* | aOR^1^ | 95% CI | *p* | OR | 95% CI | *p* | aOR^2^ | 95% CI | *p* |
|  | n=763 | | | n=708 | | | n=763 | | | n=704 | | |
| **General practitioners** | 1 |  | 0.004 | 1 |  | 0.002 | 1 |  | <0.001 | 1 |  | <0.001 |
| **Specialist doctors** | 1.96 | 0.95,4.03 |  | 1.46 | 0.62,3.42 |  | 4.90 | 2.49,9.76 |  | 3.11 | 1.40,6.92 |  |
| **Primary care nurses** | 2.01 | 0.88,4.59 |  | 2.25 | 0.85,5.95 |  | 1.48 | 0.74,2.96 |  | 2.29 | 0.99,5.30 |  |
| **Specialist nurses** | 3.23 | 1.65,6.32 |  | 2.43 | 1.05,5.62 |  | 2.86 | 1.61,5.08 |  | 1.65 | 0.76,3.57 |  |
| **Midwives** | 3.19 | 1.62,6.30 |  | 2.77 | 1.29,5.92 |  | 6.74 | 3.33,13.64 |  | 5.96 | 2.91,12.22 |  |
| **Pharmacists** | 4.56 | 2.05,10.13 |  | 6.63 | 2.56,17.13 |  | 8.12 | 3.20,20.60 |  | 14.77 | 4.65,47.02 |  |
|  | Primary care nurses | | | | | | Primary care nurses | | | | | |
|  | OR | 95% CI | *p* | aOR | 95% CI | *p* | OR | 95% CI | P value | aOR^3^ | 95% CI | *p* |
|  | n=763 | | |  | | | n=763 | | | n=710 | | |
| **General practitioners** | 1 |  | 0.007 |  |  |  | 1 |  | <0.001 | 1 |  | <0.001 |
| **Specialist doctors** | 3.00 | 1.36,6.63 |  |  |  |  | 5.5 | 2.62,11.57 |  | 4.33 | 1.86,10.05 |  |
| **Primary care nurses** | 2.62 | 1.19,5.78 |  |  |  |  | 1.15 | 0.64,2.06 |  | 1.18 | 0.62,2.25 |  |
| **Specialist nurses** | 4.03 | 1.82,8.90 |  |  |  |  | 2.93 | 1.50,5.73 |  | 2.09 | 1.02,4.28 |  |
| **Midwives** | 2.88 | 1.35,6.18 |  |  |  |  | 3.46 | 1.90,6.28 |  | 2.38 | 1.26,4.48 |  |
| **Pharmacists** | 5.57 | 2.28,13.57 |  |  |  |  | 6.75 | 2.84,16.03 |  | 7.55 | 2.81,20.28 |  |
|  | Specialist nurses | | | | | | Specialist nurses | | | | | |
|  | OR | 95% CI | *p* | aOR^4^ | 95% CI | *p* | OR | 95% CI | P value | aOR^5^ | 95% CI | *p* |
|  | n=763 | | | n=755 | | | n=763 | | | n=709 | | |
| **General practitioners** | 1 |  | 0.02 | 1 |  | 0.07 | 1 |  | 0.006 | 1 |  | 0.02 |
| **Specialist doctors** | 0.92 | 0.48,1.77 |  | 0.98 | 0.51,1.88 |  | 2.02 | 0.36,11.34 |  | 2.16 | 0.15,30.92 |  |
| **Primary care nurses** | 0.95 | 0.47,1.93 |  | 0.93 | 0.46,1.89 |  | 0.26 | 0.79,0.85 |  | 0.14 | 0.02,0.87 |  |
| **Specialist nurses** | 0.89 | 0.49,1.62 |  | 0.94 | 0.51,1.73 |  | 1.56 | 0.44,5.53 |  | 1.16 | 0.16,8.25 |  |
| **Midwives** | 0.45 | 0.25,0.82 |  | 0.5 | 0.27,0.94 |  | 1.00 | 0.33,3.03 |  | 0.42 | 0.07,2.73 |  |
| **Pharmacists** | 0.73 | 0.35,1.53 |  | 0.9 | 0.42,1.91 |  | 0.54 | 0.15,2.01 |  | 0.23 | 0.03,1.79 |  |

1.6 Crude and adjusted odd ratios for the association between type of HCP and views about which aspects of abortion care should be undertaken by midwives and nurses

^1^Adjusted for abortion provision status, age, religious beliefs, time qualified and attitudes

^2^Adjusted for abortion provision status, age, religious beliefs, time qualified, political beliefs and attitudes

^3^ Adjusted for abortion provision status, age time qualified and attitudes

^4^Adjusted for age and time qualified

^5^Adjusted for religion, time qualified, political beliefs and attitudes

P values: adjusted Wald test
